# The Dual-Language Semantic Computerized Program (DISC) Maintained Local Switch Costs in MCI Older Adults

**DOI:** 10.1093/geroni/igab046.1998

**Published:** 2021-12-17

**Authors:** W Quin Yow, Hui-Ching Chen, Tharshini Lokanathan

**Affiliations:** 1 Singapore University of Technology & Design, Singapore, Not Applicable, Singapore; 2 Singapore University of Technology and Design, Singapore University of Technology and Design, Not Applicable, Singapore

## Abstract

It has been proposed that switching cost deficit in executive control (Velichkovsky et al., 2020) could be used as an early marker for abnormal aging processes. Although research with technology-based intervention has shown benefits in improving cognitive performance with older adults, the overall results are mixed (Ge et al, 2018). This study aims to investigate whether computerized intervention program (e.g., DISC) would help to reduce the switching costs deficits in mild-to-moderate cognitively-impaired older adults (MCI-OA). Fourteen MCI-OA (79.75±6,94) and 9 cognitively-healthy OA (age 77,25±6,9) were randomly assigned to an experimental group or a control group (a final sample size of 30 MCI and 40 cognitive-healthy older adults would be ready by conference time). All participants first completed a set of cognitive tasks as part of a larger study (i.e., pre-tests) (e.g., MMSE, Ravens, cued-base Task Switching Task). The experimental group then played cognitive games on a touch-screen tablet for about 30-40 minutes per session with a total of 24 sessions over 8-12 weeks. The control group continued their daily activity as per usual for 8-12 weeks. Participants were then asked to complete the same set of cognitive tasks again post-test. Control group MCI-OA performed worse for the local costs in the cued Task Switching task (p<.05), whereas experimental group MCI-OA maintained their performance (p=.40) post-test compared to pre-test. All cognitively-healthy OA did not show any difference in performance irrespective of condition. This suggests that the DISC program could be an effective tool in slowing down the abnormal accelerated aging process.

